# A Case of Mucus Plug Removal via Bronchoscopy Using a Larger-Sized Endotracheal Tube in a Child With Plastic Bronchitis

**DOI:** 10.7759/cureus.99281

**Published:** 2025-12-15

**Authors:** Tamotsu Gotou, Takahiro Hagihara, Yamato Wada, Kyoji Hashimoto

**Affiliations:** 1 Department of Pediatric Emergency and Intensive Care Medicine, Tottori Prefectural Central Hospital, Tottori, JPN; 2 Department of Emergency and Intensive Care Medicine, Tottori Prefectural Central Hospital, Tottori, JPN

**Keywords:** bronchoscopy, child, endotracheal tube, mucus plug, plastic bronchitis

## Abstract

Plastic bronchitis is a severe condition characterised by the formation of dendritic mucus plugs within the trachea and bronchi, leading to respiratory failure due to acute airway obstruction. Bronchoscopic mucus plug removal is considered effective; however, in paediatric patients, it is often difficult to perform due to limitations in tracheal tube size. We report a case of plastic bronchitis associated with influenza A infection during a relapse of nephrotic syndrome. Bronchoscopic mucus plug removal was performed using a cuffed endotracheal tube one size larger than standard, thereby avoiding the need for extracorporeal membrane oxygenation (ECMO). The use of modified cuffed tubes with minimal outer diameter differences highlights the importance of flexible size selection tailored to the patient's condition.

## Introduction

Plastic bronchitis is a rare but potentially life-threatening condition characterized by the formation of dendritic mucus plugs within the bronchi, leading to rapid airway obstruction and resulting in atelectasis or severe respiratory failure [1]. These bronchial casts can form and enlarge over a short period, and once they obstruct a lobar or main bronchus, gas exchange deteriorates quickly. Bronchoscopic removal of mucus plugs is considered an effective treatment once the diagnosis is suspected; however, in paediatric patients, the narrow airway diameter limits the choice of endotracheal tube size and may prevent simultaneous ventilation and bronchoscope insertion. When the inner diameter of an age-appropriate tube is smaller than the minimum required for the available bronchoscope, airway management becomes technically challenging and extracorporeal membrane oxygenation (ECMO) may be required in severe cases [2]. We report a paediatric case of plastic bronchitis in which we intentionally used a cuffed endotracheal tube one size larger than the standard age-based recommendation to permit bronchoscope insertion, successfully removed the mucus plug, and achieved a favorable outcome without the need for ECMO.

## Case presentation

**Figure 1:**
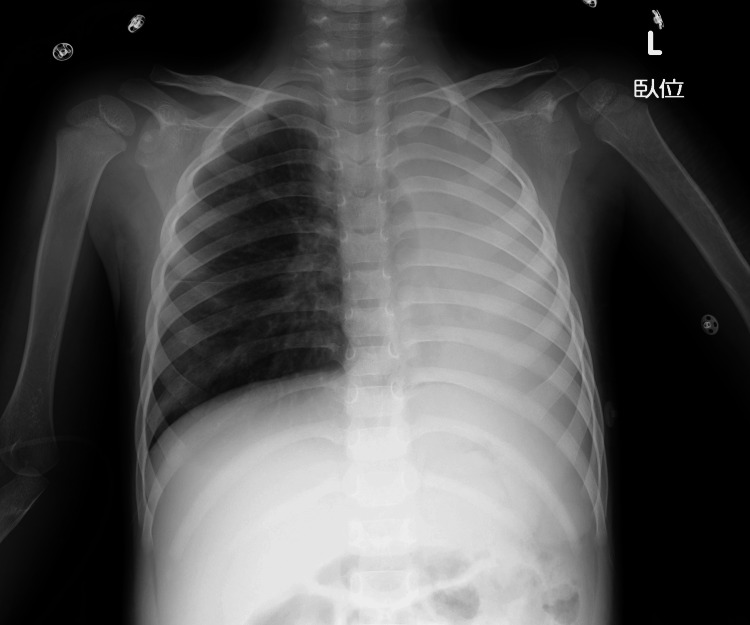
Pre-intubation chest X-ray Admission image showing total atelectasis of the left lung

The patient was a three-year-old boy. He had no history of asthma or allergies. He was hospitalised for a relapse of nephrotic syndrome and was receiving intravenous prednisolone at 2 mg/kg/day. On the eighth day of hospitalisation, he developed fever and nasal discharge and tested positive for influenza A antigen. The following day, respiratory effort increased, and SpO₂ decreased to 90% (on oxygen mask with reservoir at 10 L/min), necessitating transfer to the intensive care unit. Physical examination revealed nasal flaring and expiratory wheezing. Chest X-ray demonstrated total atelectasis of the left lung (Figure 1). 

**Figure 2:**
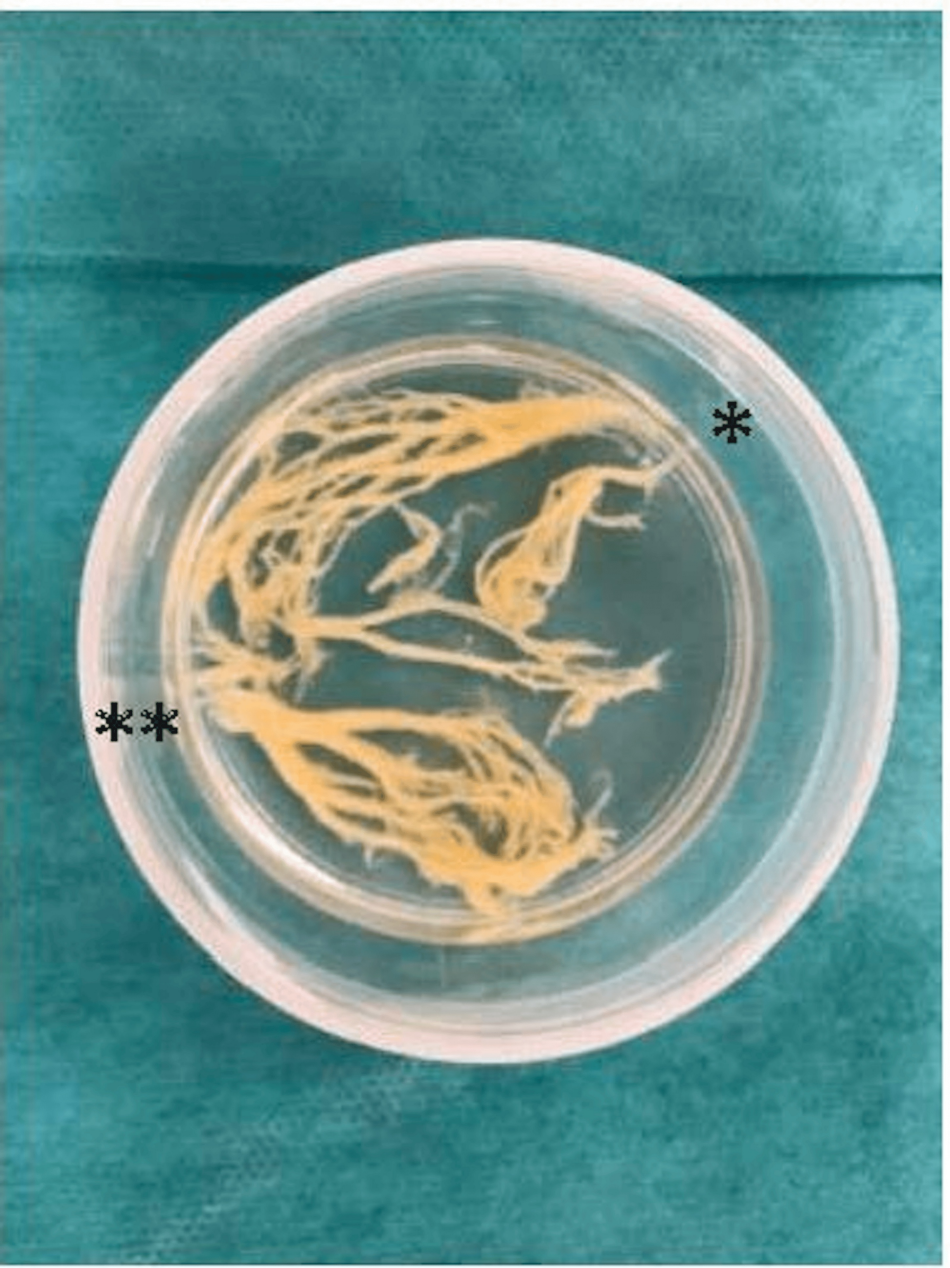
Mucus plug removed by bronchoscopy Branch-like mucus plug at the site of left main bronchus obstruction. ＊Left upper lobe branch  ＊＊Left lower lobe branch

Suspecting plastic bronchitis, endotracheal intubation was performed. The standard cuffed tube size for the patient’s age had an inner diameter of 4.5 mm, but the single-use flexible bronchoscope available at our hospital (GlideScope BFlex 3.8®, Verathon Inc., Bothell, WA, USA) has an insertion-tube outer diameter of 3.8 mm and is validated for use only with tracheal tubes with an inner diameter of at least 5.0 mm [3]. We therefore compared the actual outer diameters of an uncuffed 5.0-mm tube (Covidien™, Medtronic, Minneapolis, MN, USA) and a cuffed 5.0-mm tube (Microcuff® 5.0, Avanos Medical, Alpharetta, GA, USA) used at our institution and confirmed that the difference was small (6.7 mm vs. 6.9 mm). After explaining these device characteristics and the potential risks and benefits to the patient’s parents, we elected to perform intubation with the 5.0-mm cuffed tube. Post-intubation, bronchoscopy revealed complete obstruction of the left main bronchus, and a dendritic mucus plug was removed via suction (Figure 2).

**Figure 3:**
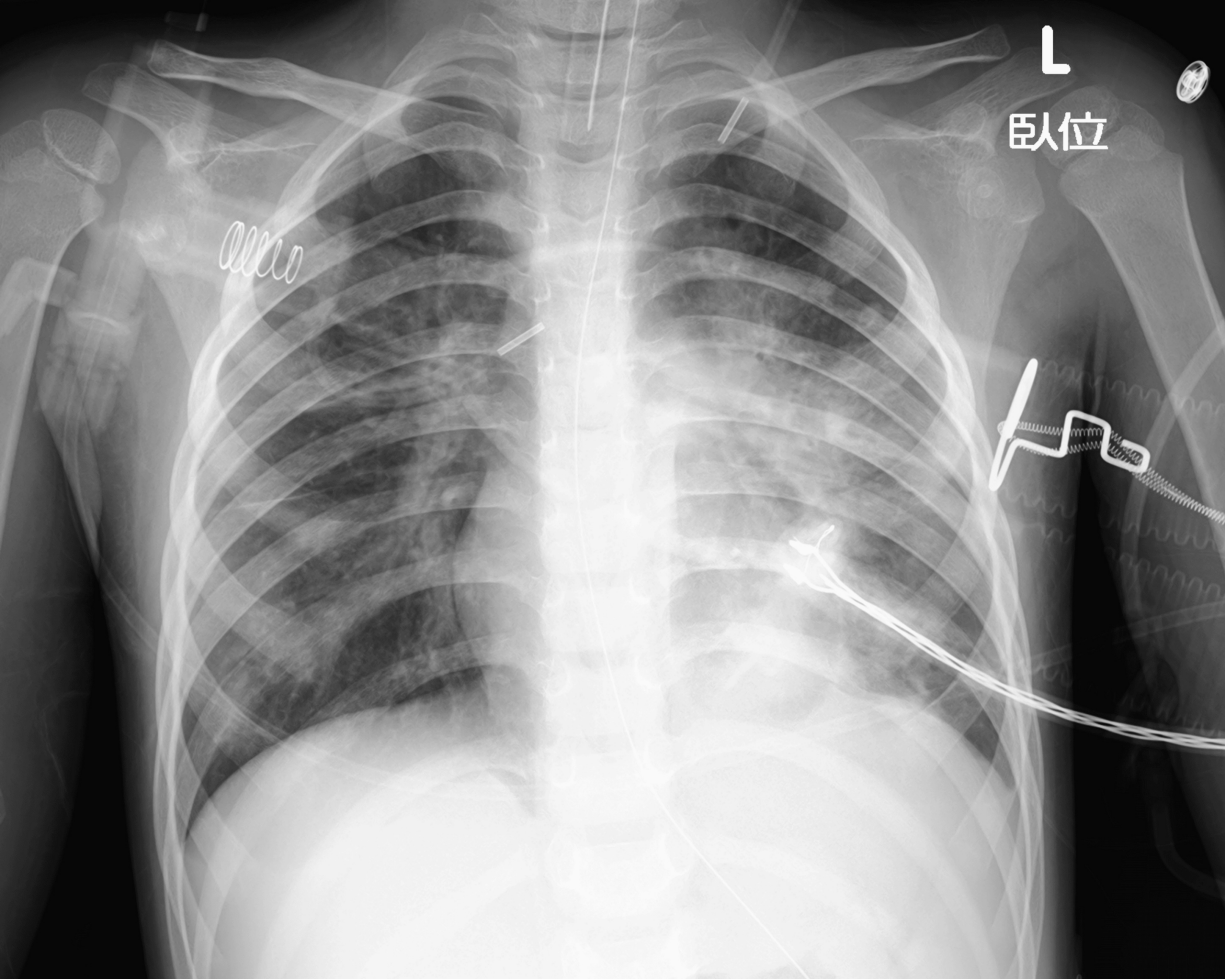
Chest X-ray after bronchoscopy The chest X-ray after removal of the mucus plug via bronchoscopy showed improvement in the atelectasis of the left lung.

The chest X-ray after removal of the mucus plug via bronchoscopy showed improvement in the atelectasis of the left lung (Figure 3).

**Figure 4:**
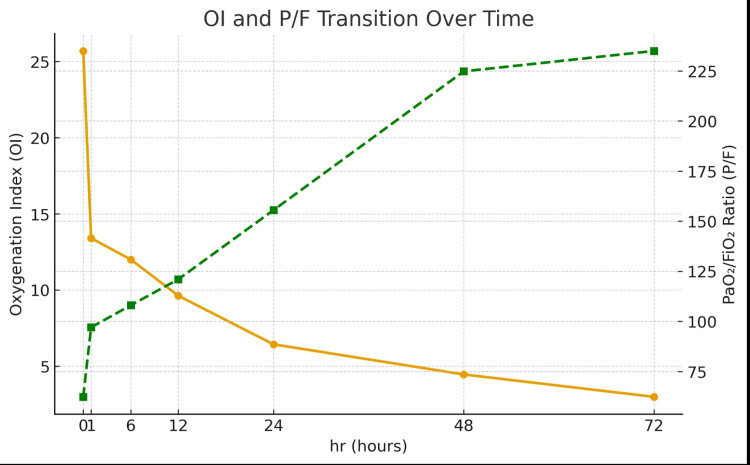
Graph showing the progression of the oxygenation index (OI) and P/F ratio This graph shows the temporal changes in the oxygenation index (OI) and PaO₂/FiO₂ ratio (P/F ratio) from intubation to 72 hours. The orange line represents the oxygenation index (OI), which is plotted on the left axis, and the green line represents the PaO₂/FiO₂ ratio (P/F ratio), which is plotted on the right axis. A rapid improvement in oxygenation was observed one hour after mucus plug removal, resulting in the patient no longer meeting the criteria for VV ECMO initiation (OI > 25, P/F < 80) [4].

Prior to mucus plug removal, the ratio of arterial oxygen partial pressure to fraction of inspired oxygen (PaO₂/FiO₂; P/F ratio) was 62.3, and the oxygenation index (OI) was 25.7, meeting our institutional criteria for venovenous extracorporeal membrane oxygenation (VV-ECMO) initiation (P/F ratio < 80 or OI ≥ 25). These thresholds are consistent with previously reported criteria for considering ECMO in severe pediatric and adult acute respiratory failure, in which very low P/F ratios and sustained elevations in OI are used to trigger ECMO evaluation [4]. One hour after mucus plug removal, the P/F ratio improved to 97 and the OI to 13.4 (Figure 4).

Subsequently, the tube was replaced with a 4.5-mm inner-diameter tube, and mechanical ventilation was continued. Extubation occurred on the 13th hospital day, and the patient was transferred to a general ward on the 15th hospital day. No peri-extubation stridor, hoarseness, or need for re-intubation was observed, and the patient remains complication-free during hospitalisation and ongoing outpatient follow-up.

## Discussion

Plastic bronchitis arises from diverse aetiologies, including infection, cardiac disease, and allergy, with some reports linking it to influenza infection. In severe cases, prompt bronchoscopic removal of bronchial casts contributes to improved prognosis; however, implementation is often challenging in paediatric patients because the tracheal tube inner diameter may not allow passage of a bronchoscope.

For decades, Cole’s age-based formula (internal diameter in mm = 4 + age/4) has been widely used to select uncuffed endotracheal tube size in children [5]. This formula was empirically derived in 1957 for uncuffed tubes using an air-leak test, at a time when outer diameters and cuff designs were not standardised across products. Subsequently, Khine and colleagues and others proposed algorithms for cuffed tubes that generally recommend choosing a cuffed tube 0.5 mm smaller than the uncuffed size predicted by Cole’s formula [6]. Subsequently, approximation formulas specific to cuffed tubes, such as the Duracher formula (ID = 3.5 + age/4), have been proposed [7].

In the late 2000s, Weiss et al. (2008) and Weber et al. (2009) reported, through multicenter collaborative studies, that the difference in outer diameter between cuffed and uncuffed tubes was extremely small. They demonstrated that the same calculation formula could be safely used for both types [8,9].

More recent clinical and experimental studies have shown that modern high-volume, low-pressure cuffed tubes, including Microcuff® and similar designs, can be used safely in children when appropriately sized and when cuff pressure is carefully monitored [10]. 

In the present case, the need to perform therapeutic bronchoscopy for suspected plastic bronchitis conflicted with standard age-based tube selection, because the available flexible bronchoscope required a minimum tracheal tube inner diameter of 5.0 mm. We therefore compared the actual outer diameters of specific cuffed and uncuffed 5.0-mm tubes from different manufacturers and confirmed that the difference in outer diameter was small. Based on this comparison, and after obtaining informed consent from the parents, we selected a cuffed 5.0-mm tube to permit bronchoscope insertion. This pragmatic, device-based decision allowed successful removal of the mucus plug and rapid improvement in oxygenation, thereby avoiding ECMO in a patient who had already reached our institutional thresholds for VV-ECMO consideration.

At the same time, this strategy carries potential risks. Intubation with an upsized cuffed tube may increase the likelihood of laryngo-tracheal injury, particularly if the tube is too tight or if cuff pressure is not adequately controlled. In our patient, there were no clinical signs suggestive of significant airway damage, such as peri-extubation stridor, hoarseness, or need for re-intubation, and the subsequent outpatient course was uneventful. Nevertheless, we did not perform systematic endoscopic evaluation of the larynx and trachea after tube exchange or at extubation, and therefore cannot exclude subtle mucosal injury. Furthermore, detailed intubation findings such as Cormack-Lehane grade, the presence and degree of leak with the cuff deflated, and serial cuff-pressure measurements were not available, which is an important limitation of this report. Accordingly, our observation should not be interpreted as evidence that upsizing cuffed tubes is generally safe in all children.

This single case highlights two practical points rather than providing definitive recommendations. First, when bronchoscopic intervention is essential in a child, careful comparison of the actual outer diameters and design characteristics of available tracheal tubes and bronchoscopes may help tailor airway management beyond simple age-based formulas. Second, even when modern high-volume, low-pressure cuffed tubes are used, clinicians should remain vigilant about potential airway trauma and should monitor cuff pressure and post-extubation airway symptoms closely. Larger case series and prospective studies are needed to clarify how device-based sizing strategies can be incorporated into paediatric airway management and whether they can be safely reflected in future guidelines.

## Conclusions

In this paediatric case of plastic bronchitis, therapeutic bronchoscopy was constrained by the minimum endotracheal tube inner diameter required for the available flexible bronchoscope. After confirming that the outer-diameter difference between selected modern cuffed and uncuffed 5.0-mm tubes was small, we intentionally used a one-size-larger cuffed endotracheal tube to facilitate bronchoscope passage. This strategy enabled prompt removal of an obstructing bronchial cast, rapid improvement in oxygenation, and avoidance of VV-ECMO initiation. The tube was subsequently exchanged to an age-appropriate size, and no clinically apparent post-extubation airway complications were observed. When urgent bronchoscopic intervention is required in children, device-specific assessment of tube and bronchoscope dimensions may support tailored airway management beyond age-based formulas; however, potential laryngo-tracheal injury risk warrants careful cuff-pressure management, close monitoring for airway symptoms, and case-by-case decision-making with informed consent.
